# Dynamics of Kv1 Channel Transport in Axons

**DOI:** 10.1371/journal.pone.0011931

**Published:** 2010-08-04

**Authors:** Yuanzheng Gu, Chen Gu

**Affiliations:** Department of Neuroscience and Center for Molecular Neurobiology, The Ohio State University, Columbus, Ohio, United States of America; INSERM U901, France

## Abstract

Concerted actions of various ion channels that are precisely targeted along axons are crucial for action potential initiation and propagation, and neurotransmitter release. However, the dynamics of channel protein transport in axons remain unknown. Here, using time-lapse imaging, we found fluorescently tagged Kv1.2 voltage-gated K^+^ channels (YFP-Kv1.2) moved bi-directionally in discrete puncta along hippocampal axons. Expressing Kvβ2, a Kv1 accessory subunit, markedly increased the velocity, the travel distance, and the percentage of moving time of these puncta in both anterograde and retrograde directions. Suppressing the Kvβ2-associated protein, plus-end binding protein EB1 or kinesin II/KIF3A, by siRNA, significantly decreased the velocity of YFP-Kv1.2 moving puncta in both directions. Kvβ2 mutants with disrupted either Kv1.2-Kvβ2 binding or Kvβ2-EB1 binding failed to increase the velocity of YFP-Kv1.2 puncta, confirming a central role of Kvβ2. Furthermore, fluorescently tagged Kv1.2 and Kvβ2 co-moved along axons. Surprisingly, when co-moving with Kv1.2 and Kvβ2, EB1 appeared to travel markedly faster than its plus-end tracking. Finally, using fission yeast *S. pombe* expressing YFP-fusion proteins as reference standards to calibrate our microscope, we estimated the numbers of YFP-Kv1.2 tetramers in axonal puncta. Taken together, our results suggest that proper amounts of Kv1 channels and their associated proteins are required for efficient transport of Kv1 channel proteins along axons.

## Introduction

Neuronal output signals are carried by concerted actions of axonal voltage-gated ion channels. These channels are precisely targeted along axons, allowing for their differential regulation of action potential initiation, waveform, uni-directional propagation and repetitive firing [1]–[5], and neurotransmitter release [6], [7]. To identify various channel motifs and interacting proteins that regulate the steady-state localization of channel complexes on neuronal membranes is an emerging research field [8]–[11]. However, the dynamics of channel protein transport within axons remain largely unknown.

The long-distance axonal transport of membrane proteins and organelles is primarily mediated by kinesin and dynein motors in anterograde and retrograde directions, respectively. Although progress has been made in understanding the roles of motor proteins in cellular function, survival and morphogenesis, how they are regulated to ensure the temporal and spatial fidelity of their functions remains to be fully elucidated [12]–[16]. Recent studies suggest that the functions of dynein and some kinesin motors can be coordinated by dynactin [17], [18].

Our previous study showed that EB1, together with KIF3A, is required for the steady-state axonal targeting of Kv1 channels, via binding to Kvβ subunits [19]. EB1 binds to the microtubule growing end autonomously, promotes microtubule extension, and enhances the fidelity of kinesin association with microtubules [20]–[23]. The link between EB1 plus-end tracking and its role in Kv1/Kvβ2 axonal targeting is not yet understood. KIF3 is a heterotrimeric complex that consists of KIF3A, KIF3B, and kinesin superfamily-associated protein 3 (KAP3). It is involved in the axonal transport of vesicles containing fodrin, a KAP3-interating protein [24], [25]. Kvβ2 regulates Kv1 channel activity, and promotes its forward trafficking and axonal targeting [26]–[28]. By binding to Kv1 cytoplasmic T1 domains, Kvβ2 may link the Kv1 complex to EB1 and KIF3 as an adaptor protein. However, the functions of these three Kv1-associated proteins in the dynamic trafficking of the channel complex remain unknown.

In this study, we visualized the bi-directional movement of YFP-Kv1.2 along axons using live-cell imaging techniques. YFP-Kv1.2 moving puncta were observed from the axon initial segment to the distal region of axons. Co-expressed Kvβ2 enhanced the travel time, the distance, and the velocity of these puncta in both directions, likely through its associated proteins, EB1 and KIF3A. Unlike many other EB1-binding proteins, neither Kvβ2 nor Kv1.2 displayed the plus-end tracking. Finally, using quantitative microscopy, we estimated the number of YFP-Kv1.2 molecules in different puncta containing YFP-Kv1.2.

## Materials and Methods

### cDNA constructs

YFP-Kv1.2, CFP-Kv1.2, YFP-Kvβ2, CFP-Kvβ2, EB1-YFP, EB1-CFP, YFP-Kvβ2K235E, YFP-Kvβ2C2, and KIF3A-YFP were previous described [19]. CFP-Kvβ2K235E was made by Quickchange mutagenesis based on CFP-Kvβ2. mCherry was a kind gift from Dr. Roger Tsien.

### Hippocampal neuron culture and transfection

Hippocampal neuron culture was prepared as previously described from E18 rat embryos [19]. In brief, 2 days after neuron plating, 1 µM cytosine arabinose (Sigma) was added to the neuronal culture medium to inhibit glial growth for the subsequent 2 days, then replaced with the normal culture medium. The culture medium was replenished twice a week by replacing half the volume. For transient transfection, neurons in culture at 5–7 DIV (*day in vitro*) were incubated in Opti-MEM containing 0.8 µg of cDNA plasmid and 1.5 µl of Lipofectamine2000 (Invitrogen) for 20 min at 37°C.

### Immunostaining and quantification

The immunostaining procedure was described previously [19]. In the present study, transfected neurons were fixed with 4% formaldehyde (methanol free) and stained at 9 DIV. This fixation and staining method should reveal total EB1 molecules, in contrast to the cold methanol method, which only reveals EB1 molecules binding to the cytoskeleton. This is because methanol fixation precipitates the proteins on the cytoskeleton and permeabilizes the cells at the same time. This is a method frequently used for visualizing microtubule-binding proteins. However, highly soluble proteins (including unbound and free EB1) will be extracted and undetected by this method. In contrast, formaldehyde fixation crosslinks proteins through free amino groups and thus immobilizes all EB1 molecules including the free ones. Antibodies used include mouse anti-EB1 antibody (BD Transduction Laboratories, San Jose, CA), rabbit polyclonal anti-KIF3A antibody (Abcam, Cambridge, MA), Cy2 and Cy5 conjugated secondary antibodies (Jackson ImmunoResearch, West Grove, PA).

Fluorescence images were captured with a Spot CCD camera RT slider (Diagnostic Instrument Inc.) in a Zeiss upright microscope, Axiophot, using Plan Apo objectives 20**×**/0.75 and 100**×**/1.4 oil, saved as 16-bit TIFF files, and analyzed with NIH Image J and Sigmaplot 11 for fluorescence intensity quantification. Exposure times were controlled so that the pixel intensities in dendrites and axons were below saturation, but the same exposure time was used within each group of an experiment. The quantification procedure was described previously [28]. Only transfected neurons with clearly separated dendrites and axons, and isolated from other transfected cells, were chosen for analysis. To distinguish axons from dendrites, axonal marker Tau1 and dendritic marker MAP2 were used for co-staining.

### siRNA knockdown of endogenous EB1 and KIF3A

Construction and validation of vector-based siRNA probes to suppress the endogenous EB1 or KIF3A levels in rat hippocampal neurons were previous described [19]. Two probes, EB1 siR2 (CAATGAATCTCTGCAGTTGAA) and KIF3A siR2 (CAAGGAAAGACCAAGAAATTA) subcloned into the pRNAT-H1.1/neo vector (GenScript), effectively suppressed endogenous EB1 and KIF3A, respectively [19]. The GFP coding sequence in the vector was replaced with the coding sequence for mCherry between BamHI and HindIII sites. Thus, the neurons transfected with the siRNA plasmid expressed mCherry as the indicator for transfection.

### Live cell FRAP imaging and two-color time-lapse imaging

Neurons growing on 25 mm coverslips were loaded into the imaging chamber (Molecular Devices, Downingtown, PA) and incubated with imaging buffer (HE-LF medium (Brainbits, Springfield, IL) plus 2% B-27, 0.5 mM Glutamine, and 25 µM Glutamate). The timelapse imaging setup was built upon a Nikon (Nikon Inc., Melville, NY) TE2000 inverted microscope. The following procedure was used to identify axons and the orientations. (1) The neurons with clearly separated axons and dendrites were selected for imaging. (2) Different from dendrites, axons were long fibers with largely consistent diameter within the same branch. (3) We moved the lens along the axon to trace its origin (neuron soma) and/or its ending (axonal growth cone) to determine the orientation.

The FRAP imaging significantly enhanced our visualization of mobile YFP-Kv1.2 puncta. First, the YFP fluorescence of immobile YFP-Kv1.2 molecules along an axonal segment (50∼150 µm) was bleached with maximal excitation lights through the diaphragm in the minimal position for 5 to 10 min. Next, the excitation lights were reduced to 12.5% of the maximal with the 8× neutral density filter and the diaphragm was fully opened. Then, we performed time-lapse imaging for mobile YFP-Kv1.2 moving into this bleached region. Images were captured with a CCD camera Coolsnap HQ (Photometrics, Tucson, AZ) through an YFP filter set with 1 second exposure time and 2 second interval for 100 frames. To obtain kymographs from stacks of original images, we drew lines with 4-pixel width along axonal segments.

In two-color time-lapse imaging, images were captured through CFP or YFP filter sets with 1-second exposure time. The filters were changed through filter wheels controlled through Lamda 10–3 (Sutter Instrument, Novato, CA) by the MetaMorph software (Molecular Devices). The TexRed filter set was used for checking mCherry expression. Kymographs from the two-color imaging were obtained with stacks of merged images.

### Quantification of mobile puncta along axons

All measurements were carried out on kymographs made with the MetaMorph program. The total time for each movie is 198 seconds. First, the travel distance (*d*), the moving time (*t_m_*), and the stationary time (*t_s_*) were measured for each punctate. Mobile puncta (*n*) were manually counted in each kymograph. Next, based on those parameters, we calculated the travel velocity (*v* = *d*/*t_m_*), the percentage of moving time *P_moving_* (%)  =  *t_m_*/(*t_m_* + *t_s_*) ×100, and the frequency of transport events (or the average number of moving puncta per movie) *F*(#/min)  =  *n*/(198 sec/60 sec/min). For instance, if one moving punctum (n = 1) is observed in one movie (total 3.3 minute long), *F* equals to 0.3. *F* equals to 0 if no moving punctum was seen in a movie, 0.6 if 2 moving puncta were seen in a movie, and 0.9 if 3 moving puncta were seen in a movie. An example for the measurement and calculation was provided in Fig. S1D. Anterograde and retrograde puncta were measured in two separate groups.

### Using fission yeast as standards to count YFP-Kv1.2 proteins in axonal puncta

We used five yeast strains (wild type and the ones expressing Arc1-mYFP, Arp2-mYFP, Fim1-mYFP, and Spn4-mYFP) [29], [30] to calibrate our quantitative microscope system. Yeast cells were grown in rich liquid medium YE5S (980 ml dd H_2_O, 5 g Difco Yeast Extract, 30 g dextrose, 225 mg/liter each of the 5 supplements adenine, uracil, leucine, histidine, and lysine, in 1 liter) at 25°C for 36 hours in a shaker (200 rpm). The cells in cultures at OD_595_ = 0.1−0.5 (2−10×10^6^ cells/ml) were used for microscopy. Yeast cells were loaded onto the coverslips used for culturing hippocampal neurons. The exact same settings for imaging YFP-Kv1.2 in hippocampal axons, including the neutral density filter, the 100× oil lens, the YFP filter set, camera, and the exposure time, were used for yeast cells. For each yeast strain, five images were randomly captured and at least ten patches or rings were measured in each image. Images captured from the wildtype yeast were used as the background for subtraction. The total fluorescence intensities (***F_total_***) in these structures were measured with the MetaMorph program. ***F_total_***  =  ***F_average_*** × the size of the area of interest. ***F_average_*** is the average YFP fluorescence intensity per pixel in the area of interest.

In our microscope system, the ***F_total_***s (mean ± SD) were 1063±357 AU (Arc1-mYFP), 1556±499 AU (Arp2-mYFP), 2394±867 AU (Fim1-mYFP), and 3425±877 AU (Spn4-mYFP). The numbers of YFP-fusion proteins (***n***) in these structures were 208±79 (Arc1-mYFP), 212±94 (Arp2-mYFP), 507±290 (Fim1-mYFP), and 6100±1200 (Spn4-mYFP) [29]. Therefore, the fluorescence intensities measured in our microscope system were largely in a linear relationship with the published numbers except Spn4-mYFP [29]. Different from other three proteins, Spn4-mYFP localizes in contractile rings, which are up to 5 µm in diameter. One frame image may not capture the majority of the fluorescence in a ring. Therefore, the fluorescence intensity obtained from our microscope system may underestimate the fluorescence intensity for Spn4-mYFP, which is consistent with our results. On the other hand, Arc1-mYFP, Arp2-mYFP, and Fim1-mYFP are in small actin patches, similar to YFP-Kv1.2 puncta in axons in size. Therefore, these three proteins were used to obtain the linear regression curve in SigmaPlot 11. ***F_total_*** = 4.95× ***n*** +61. R^2^ = 0.96. In the regression, four points with 0 (***F_total_***) and 0 (***n***) were added to force the curve to get close to the zero point. Fluorescence intensities of YFP-Kv1.2 puncta were measured. The estimated number of YFP-Kv1.2 molecules (***n***) and the number of channel tetramers (***n***/4) were calculated for each punctum.

## Results

### Kvβ2 enhanced the axonal transport of YFP-Kv1.2 in both anterograde and retrograde directions

**Figure 1:**
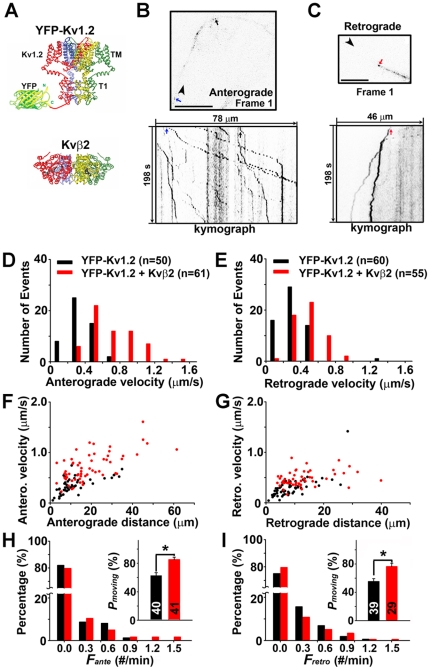
Kvβ2 enhances both anterograde and retrograde transport of YFP-Kv1.2 puncta along hippocampal axons. (A) Structural diagrams of YFP-Kv1.2 and Kvβ2. This Kv1.2 channel tetramer includes intracellular N-terminal T1 domains (T1) and membrane-spanning segments (TM), but not C-terminal domains. For clarification, only one YFP molecule is shown to be fused to the N-terminus of the red subunit. Kvβ2 also exists as tetramers. (B) The first frame (top) and kymograph (bottom) of anterograde movement of YFP-Kv1.2 puncta in the presence of co-expressed Kvβ2. (C) The first frame (top) and kymograph (bottom) of retrograde movement of YFP-Kv1.2 puncta. Blue, black and red arrows, three moving puncta pointed out in both the first frame and kymograph. Black arrowheads, the direction of axonal terminals. Co-expressed Kvβ2 increased the velocity of YFP-Kv1.2 puncta in both anterograde (D) and retrograde (E) directions. Expressed Kvβ2 altered the velocity and distance relationship in both anterograde (F) and retrograde (G) directions. The frequency of transport events and the percentage of moving time of puncta are provided for anterograde (H) and retrograde (I) directions. The number of puncta in each condition is provided in the bar. *, *p*<0.05 in *t* test.

To visualize the movement of axonal Kv1 channels, we transfected YFP-Kv1.2 (Fig. 1A) into cultured hippocampal neurons and performed live-cell imaging. Expressed YFP-Kv1.2 smoothly distributed along axons, hindering the visualization of its movement. Thus, we adopted a fluorescence recovery after photobleaching (FRAP) strategy. The YFP fluorescence of YFP-Kv1.2 along an axonal segment (50∼150 µm) was bleached first, and then unbleached YFP-Kv1.2 moving into this region was visualized with time-lapse imaging (Figure S1A). YFP-Kv1.2 moved in both anterograde and retrograde directions in discrete puncta (Fig. 1B,C). The anterograde puncta moving towards axonal endings were all tiny and vesicular shaped (Fig. 1B; Movie S1); the retrograde puncta moving towards the soma were slightly bigger and significantly slower than the anterograde ones (Fig. 1C; Movie S2).

To assess how Kvβ2 might affect the dynamics of YFP-Kv1.2 puncta in axons, we compared the behavior of these puncta in the absence and presence of co-expressed Kvβ2 (Figure S1B-D). In the absence of co-expressed Kvβ2, anterograde travel velocities of YFP-Kv1.2 puncta ranged from 0 to 0.8 µm/s (average anterograde velocity: 0.35±0.02 µm/s, n = 50) (Fig. 1D). The velocity distribution significantly shifted to the right in the presence of co-expressed Kvβ2 (average anterograde velocity: 0.71±0.04 µm/s, n = 61) (Fig. 1D). In contrast to the expected enhancement of YFP-Kv1.2 anterograde transport by Kvβ2, it was a surprise that co-expressed Kvβ2 also significantly increased the retrograde velocity (average retrograde velocity: control, 0.30±0.02 µm/s, n = 60; + Kvβ2, 0.48±0.02 µm/s, n = 55; p<0.001 in *t* test) (Fig. 1E). Moreover, in the presence of expressed Kvβ2, both the anterograde (control, 11.5±1.2 µm, n = 50; + Kvβ2, 19.0±1.5 µm, n = 61; p<0.001 in *t* test) and retrograde (control, 8.7±0.7 µm, n = 60; + Kvβ2, 13.1±1.0 µm, n = 55; p<0.05 in *t* test) travel distances of YFP-Kv1.2 puncta significantly increased (Fig. 1F,G).

In the presence of co-expressed Kvβ2, the frequency of moving puncta containing YFP-Kv1.2 increased in the anterograde (***F_ante_***) direction but not in the retrograde (***F_retro_***) direction, while the majority (∼70–80%) of the movies did not contain any moving punctum (Fig. 1H,I). The percentage of moving time (***P_moving_***) of YFP-Kv1.2 puncta significantly increased in both anterograde (control, 62.6±4.2%, +Kvβ2, 85.3±3.4%; p<0.01) and retrograde (control, 55.3±4.0%, +Kvβ2, 76.4±4.7%; p<0.01) directions (Fig. 1H,I). Furthermore, we visualized the movement of YFP-Kv1.2 puncta in different axonal regions, from the proximal (including axon initial segments) to distal (including axonal growth cones) segments of axons, and found no major difference in moving dynamics (Figure S1B,C).

### Suppressing endogenous EB1 or KIF3A markedly reduced YFP-Kv1.2 axonal transport

**Figure 2:**
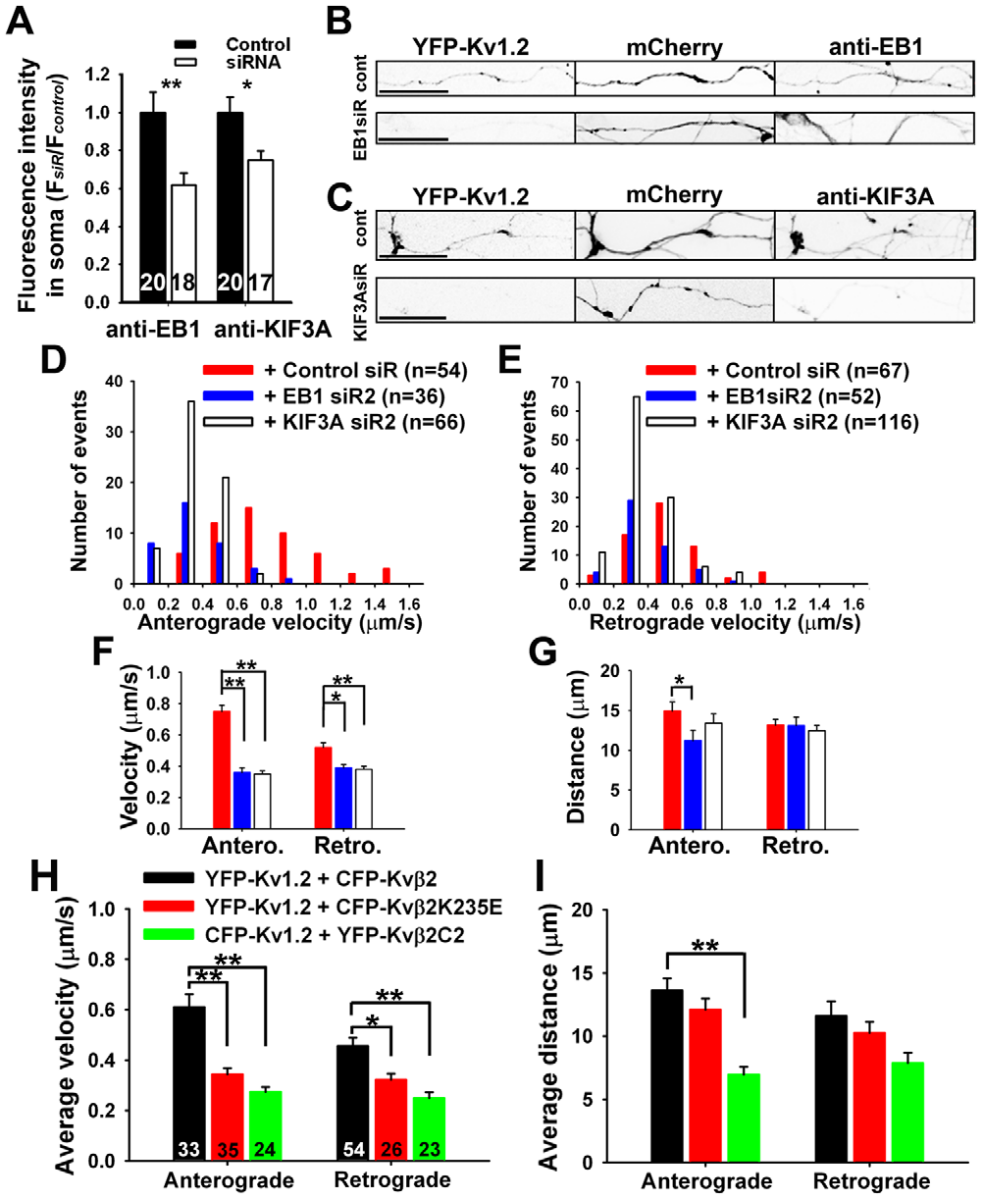
Suppressing endogenous EB1 and KIF3A reduces the dynamics of YFP-Kv1.2 puncta. (A) Vector-based siRNA significantly suppressed the levels of endogenous EB1 and KIF3A. The vectors contain mCherry as the transfection indicator. The fluorescence intensities of EB1 and KIF3A in the soma of transfected neurons were measured. Hippocampal neurons co-transfected at 4 DIV with YFP-Kv1.2, Kvβ2, and siRNA plasmids against either EB1 (B) or KIF3A (C) were fixed and stained 4–5 days later. Reduction of either EB1 (B) or KIF3A (C) decreased the axonal level of YFP-Kv1.2. The live cell imaging was carried out 4–5 days after co-transfection. (D) Distribution of anterograde travel velocities in the presence of control siRNA (red), EB1 siR2 (blue), or KIF3A siR2 (white). (E) Distribution of retrograde velocities. (F) Effects of siRNA knockdowns on average velocities of YFP-Kv1.2 puncta. (G) Effects of siRNA knockdowns on average travel distances. (H) Effects of Kvβ2 mutants on the average velocity of Kv1.2 axonal transport. YFP-Kv1.2 was co-transfected with either CFP-Kvβ2 (black) or CFP-Kvβ2K235E (red) into hippocampal neurons. To examine the effect of the Kvβ2 mutant that fails to bind to EB1, we co-transfected CFP-Kv1.2 and YFP-Kvβ2C2 (green) into hippocampal neurons. The number within each bar is “n”. (I) Effects of Kvβ2 mutants on the average travel distance of Kv1.2 axonal transport. Bar graphs are presented in mean ± SEM. *, *p*<0.05; **, *p*<0.001 in *t* test.

Based on our previous study [19], we hypothesized that Kvβ2 enhanced YFP-Kv1.2 axonal transport via EB1 and KIF3A. To determine how EB1 and KIF3A might affect the dynamics of YFP-Kv1.2 axonal transport, we knocked down endogenous EB1 and KIF3A by vector-based siRNA. We modified the siRNA plasmids made previously by replacing the GFP with mCherry as the indicator for transfection. Four days after transfection, the corresponding siRNA plasmid significantly suppressed the endogenous EB1 (control siR: 1.00±0.11; EB1 siR2: 0.62±0.07) or KIF3A (control siR: 1.00±0.08; EB1 siR2: 0.75±0.05) levels at the soma (Fig. 2A; Figure S2), consistent with our previous study [19]. Next, we co-transfected neurons with YFP-Kv1.2, Kvβ2, and either control siRNA, EB1 siR2, or KIF3A siR2. The transfected neurons were stained for endogenous EB1 or KIF3A. Suppressing either EB1 or KIF3A reduced the axonal level of YFP-Kv1.2 (Fig. 2B,C; Figure S3).

The axons expressing both YFP and mCherry fluorescence were chosen for the FRAP imaging of YFP-Kv1.2. Suppressing endogenous EB1 markedly reduced the anterograde (control siR: 0.75±0.04 µm/s; EB1 siR2: 0.36±0.03 µm/s) and retrograde (control siR: 0.52±0.03 µm/s; EB1 siR2: 0.39±0.02 µm/s) velocities of YFP-Kv1.2 puncta (Fig. 2D–F). The anterograde travel distance also significantly shortened (Fig. 2G). Similarly, suppressing endogenous KIF3A significantly reduced the anterograde (KIF3A siR2: 0.35±0.02 µm/s) and retrograde (KIF3A siR2: 0.38±0.02 µm/s) velocities of YFP-Kv1.2 puncta (Fig. 2D–F). Moreover, suppressing EB1 or KIF3A significantly reduced the frequency of anterograde YFP-Kv1.2 puncta but did not change the percentage of moving time (data not shown). Taken together, suppressing either endogenous EB1 or KIF3A level significantly reduced velocities of YFP-Kv1.2 in both anterograde and retrograde directions, which is exactly opposite to the effect of co-expressed Kvβ2.

### Effects of Kvβ2 mutations on YFP-Kv1.2 axonal transport

Our results suggest a central role of Kvβ2 in regulating Kv1.2 axonal transport. Kvβ2 may link Kv1.2 channel via the T1-Kvβ2 interaction, to the axonal transport machinery via the Kvβ2-EB1 interaction. To determine whether the T1-Kvβ2 interaction is important for enhancing axonal transport of YFP-Kv1.2 in the presence of co-expressed Kvβ2, we examined the effect of a point mutation (K235E) of Kvβ2 that eliminates the T1-Kvβ2 binding [19]. This residue is located in the T1-kvβ2 interface. The point mutation eliminates the effect of Kvβ2 on Kv1.2 membrane targeting and axonal targeting via disrupting the Kv1.2-Kvβ2 binding, although the mutant itself is still concentrated in axons [19]. In this study, either CFP-Kvβ2 or CFP-Kvβ2K235E was co-transfected with YFP-Kv1.2 into hippocampal neurons and their effects on YFP-Kv1.2 axonal transport were compared. In the presence of CFP-Kvβ2K235E, both the anterograde (0.34±0.03 µm/s) and retrograde velocity (0.32±0.02 µm/s) of YFP-Kv1.2 puncta significantly decreased, compared to CFP-Kvβ2 (anterograde: 0.61±0.05 µm/s; retrograde: 0.46±0.03 µm/s) (Fig. 2H). Travel distances of YFP-Kv1.2 puncta were similar in the presence of either CFP-Kvβ2 or CFP-Kvβ2K235E (Fig. 2I).

Next, we examined how interfering Kvβ2-EB1 binding affects Kv1 axonal transport. In our previous study, we identified a mutant of Kvβ2 (YFP-Kvβ2C2) whose binding to EB1 was disrupted [19]. YFP-Kvβ2C2 itself was restricted in the somatodendritic region [19]. When co-expressed with YFP-Kvβ2C2, the axonal level of CFP-Kv1.2 was low, compared to the co-expression with Kvβ2 (data not shown). In the presence of YFP-Kvβ2C2, average anterograde (0.27±0.02 µm/s) and retrograde (0.25±0.02 µm/s) velocities of CFP-Kv1.2 puncta were significantly lower than those of YFP-Kv1.2 puncta in the presence of CFP-Kvβ2 (Fig. 2H). Furthermore, the anterograde (6.95±0.62 µm), but not retrograde, travel distance in the presence of YFP-Kvβ2C2 was significantly less, compared to CFP-Kvβ2 (13.61±0.95 µm) (Fig. 2I). Taken together, our mutagenesis results support a central role of Kvβ2 in Kv1.2 axonal transport.

### Increased velocity of EB1 when co-moving with Kv1.2 or Kvβ2

**Figure 3:**
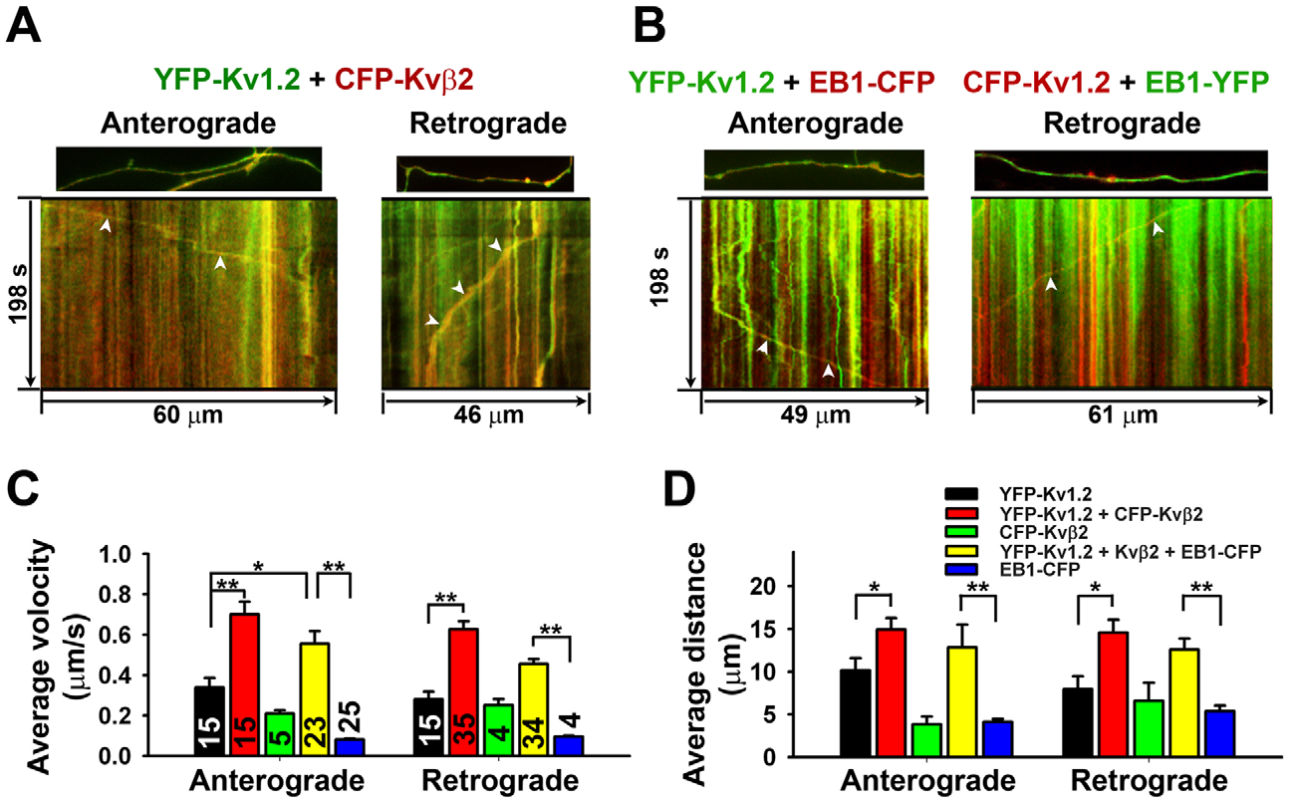
Kv1.2 co-moves with Kvβ2 and EB1 along axons. Hippocampal neurons were transfected at 5 DIV and imaged 2–3 days later. (A) YFP-Kv1.2 (green) co-moved with CFP-Kvβ2 (red) in both anterograde and retrograde directions. The first frame (top) and kymograph (bottom) are provided. (B) YFP-Kv1.2 co-moved with EB1-CFP in the anterograde direction (left). CFP-Kv1.2 and EB1-YFP co-moved in the retrograde direction (right). White arrowheads, co-movements in kymographs. (C) Average travel velocity for puncta containing YFP-Kv1.2 alone (black), both YFP-Kv1.2 and CFP-Kvβ2 (red), CFP-Kvβ2 alone (green), both YFP-Kv1.2 and EB1-CFP in the presence of Kvβ2 (yellow), or EB1-YFP alone (blue). (D) Average travel distance. The numbers within the bars, n, are the same for (C) and (D). *, *p*<0.05; **, *p*<0.001 in *t* test.

Kv1 associated proteins could regulate the mobility of Kv1 puncta via different mechanisms. They may co-assemble with Kv1 channels in the same punctum and directly regulate the activity of associated motor proteins. Alternatively, they may not need to co-move with the channels, but regulate microtubule and associate proteins to indirectly regulate the motor activity. To determine whether and how Kv1.2 and its associated proteins move together, we performed two-color time-lapse imaging. First, we imaged the axons expressing both YFP-Kv1.2 and CFP-Kvβ2. The two highly colocalized along axons. Whereas most puncta were stationary, we observed mobile puncta containing both YFP-Kv1.2 and CFP-Kvβ2 moving in either anterograde or retrograde direction (Fig. 3A), as well as mobile puncta containing only one of them. The velocities and travel distances of these puncta were measured. Interestingly, the puncta containing both YFP-Kv1.2 and CFP-Kvβ2 moved markedly faster in the anterograde direction (0.70±0.06 µm/s), compared to those containing only one (YFP-Kv1.2 only: 0.34±0.05 µm/s; CFP-Kvβ2 only: 0.21±0.02 µm/s) (Fig. 3C). This was also the case in the retrograde direction (Fig. 3C). The puncta containing both also moved over longer distances compared to those containing only one (Fig. 3D). This is consistent with the result that co-expressed Kvβ2 significantly increased the velocity and travel distance of YFP-Kv1.2 puncta in both directions (Fig. 1).

This result raised an intriguing question. The average velocity of YFP-Kv1.2 puncta in the presence of co-expressed Kvβ2 was approximately 0.75 µm/s, whereas the average velocity of EB1 plus-end tracking was only around 0.1 µm/s. How could EB1 help the anterograde transport of Kv1.2/Kvβ2 complex if they move at different velocities?

To determine whether and how Kv1.2 and EB1 may co-move, we imaged neurons co-transfected with Y(C)FP-Kv1.2, Kv***β***2, and EB1-C(Y)FP. Interestingly, the fluorescently-tagged Kv1.2 and EB1 also co-moved in both anterograde and retrograde directions (Fig. 3B). Surprisingly, they co-moved at a much faster velocity in the anterograde direction (average velocity: 0.56±0.06 µm/s) compared to the puncta containing only EB1-CFP (average velocity: 0.08±0.01 µm/s, plus-end tracking) (Fig. 3C). In the retrograde direction, puncta containing both also moved faster (Fig. 3C). Approximately, one third of mobile puncta contained both Kv1.2 and EB1, and those puncta traveled over longer distances in both directions (Fig. 3D). Therefore, when co-moving together with Kv1.2, EB1 displayed the moving behavior drastically different from its well-known plus-end tracking.

To determine whether the velocity change occurs when Kvβ2 and EB1 co-move, we performed the time-lapse imaging on neurons co-transfected with CFP-Kvβ2 and EB1-YFP. Compared to the plus-end tracking of EB1-YFP, the puncta containing both CFP-Kvβ2 and EB1-YFP moved faster in both directions (Figure S4A–D). Therefore, neither Kv1.2- nor Kvβ2-containing puncta displayed the plus-end tracking when co-moving with EB1.

To assess whether Kv1.2-containing puncta co-moves with the KIF3 motor, we co-transfected neurons with KIF3A-YFP, CFP-Kv1.2 and Kvβ2, and performed two-color time-lapse imaging. KIF3A-YFP and CFP-Kv1.2 highly colocalized. While most puncta were stationary, mobile puncta containing both of them were observed in both anterograde and retrograde directions (Figure S4E). There was no significant difference in velocities and travel distances among puncta either containing both KIF3A-CFP and YFP-Kv1.2 or containing only one of them (Figure S4E). Therefore, increasing the expression level of either EB1 or KIF3A in axons did not significantly increase the velocity of YFP-Kv1.2 puncta (Fig. 3C; Figure S4E).

### The numbers of YFP-Kv1.2 channel tetramers within different YFP-Kv1.2 puncta

**Figure 4:**
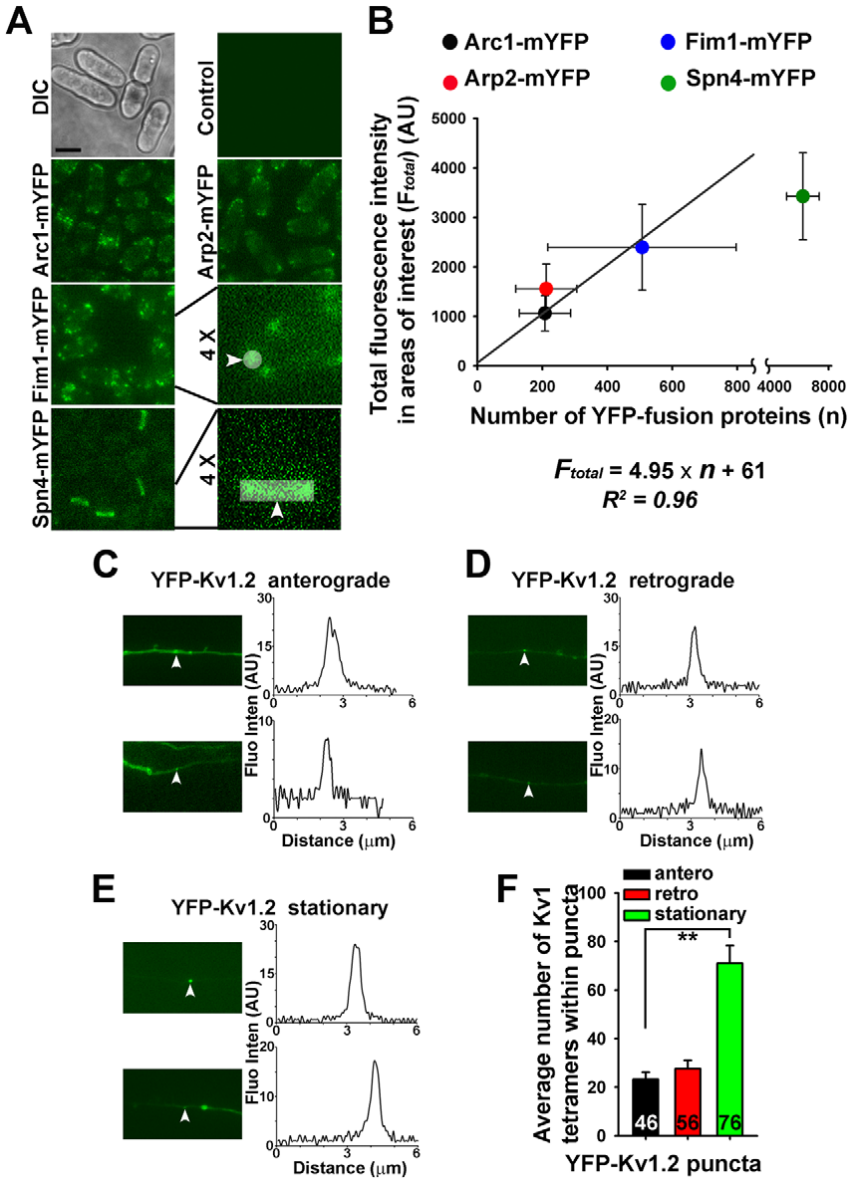
Estimating the number of YFP-Kv1.2 channel tetramers in different transporting vesicles along hippocampal axons. (A) Images of yeast cells. Control cells and the cells expressing Arc1-mYFP, Arp2-mYFP, Fim1-mYFP, and Spn4-mYFP were imaged. Images with 4-fold higher magnification for Fim1-mYFP and Spn4-mYFP are provided on the right. White arrowheads point to the areas of interest. (B) The relationship between the fluorescence intensity (mean ± SD) of actin patches imaged from our microscope and published numbers of YFP-fusion proteins. A simple linear regression curve was generated. Examples of anterograde moving (C), retrograde moving (D), and stationary (E) puncta (indicated by white arrowheads) of YFP-Kv1.2 along axons are provided. Axonal segments are given on the left, and the fluorescence profile along a 6 µm line across the punctum is given on the right. (F) Average numbers of Kv1 channel tetramers in anterograde (antero, black), retrograde (retro, red), and stationary (green) puncta. **, *p*<0.001 in *t* test.

The efficiency of Kv1 channel targeting depends on not only the velocity, the travel distance and the frequency of transporting vesicles, but also the number of channel proteins within each vesicle. To estimate the numbers of YFP-Kv1.2 channels in these puncta, we calibrated our quantitative fluorescence microscopy using yeast *S. pombe* expressing different YFP-fusion proteins. The numbers of YFP-fusion proteins in these cells were previous determined [29]. We first imaged five different yeast cells, one wildtype and four expressing different YFP-fusion proteins (Fig. 4A). Consistent with the previous results [29], Arc1-mYFP, Arp2-mYFP, and Fim1-mYFP were concentrated in small actin patches (<1 µm in diameter), whereas Spn4-mYFP was concentrated in contractile rings (Fig. 4A). Fluorescence intensities of the first three were measured and were largely in a linear relationship with the published numbers of YFP-fusion proteins in actin patches [29]. A simple linear regression curve was generated between the total fluorescence intensity and the number of YFP-fusion proteins (Fig. 4B).

Next, we measured the fluorescence intensity of every YFP-Kv1.2 punctum that moved either anterogradely or retrogradely, or was simply stationary (Fig. 4C–E). Using the linear regression equation (***F_total_*** = 4.95× ***n*** +61), we calculated the number of YFP-Kv1.2 molecules (***n***) in each punctum. Since Kv1 channels are tetramers, a YFP-Kv1.2 tetrameric channel should contain four YFP-Kv1.2 molecules. Therefore, the number of YFP-Kv1.2 channel tetramers in each punctum equals to ***n***/4. Our data show that the numbers of channel tetramers in YFP-Kv1.2 puncta varied (Fig. 4F). There were on average 23 YFP-Kv1.2 tetramers in anterograde puncta (23.2±3.0), only slightly less than those in retrograde ones (27.5±3.6), but significantly less than those in stationary ones (71.0±7.3) (Fig. 4F).

## Discussion

In this study, we visualized the dynamic movement of Kv1.2 channels along axons using live-cell imaging techniques. YFP-Kv1.2 resided in round puncta in axons, moving bi-directionally at different velocities (Fig. 1). Kv1 accessory subunit, Kvβ2, and its associated proteins, EB1 and KIF3A, profoundly regulate the dynamics of these moving puncta (Figs. 2 and 3).

Our data suggest that Kvβ2 may not only enhance the loading of Kv1 cargos onto the kinesin motors, but also stimulate the motor activities. Co-expressed Kvβ2 significantly increased the travel distance and the percentage of moving time of YFP-Kv1.2 puncta, and increased the frequency of anterogradely-moving puncta (Fig. 1F,H). These results are consistent with the notion that Kvβ2 may link Kv1 channel complex to the motors. Interestingly, co-expressed Kvβ2 markedly increased the velocity of YFP-Kv1.2 puncta moving in the anterograde direction (Fig. 1D), suggesting that it also stimulated the activity of the motors. This is consistent with the result that CFP-Kvβ2 within the YFP-Kv1.2 punctum increased its velocity (Fig. 3A,C), presumably through a direct action on the kinesin motors. Kvβ2 is expressed in young neurons, but at a low level [19], so that the increased level of Kvβ2 significantly enhanced the transport of YFP-Kv1.2 puncta.

The endogenous levels of EB1 and KIF3A appeared to be sufficient to transport Kv1 channel complexes, since over-expression of the two did not further enhance the YFP-Kv1.2 transport (Fig. 3; Figure S4E). In contrast, even a partial knockdown of EB1 and KIF3A levels had a major effect on YFP-Kv1.2 axonal transport (Fig. 2). Suppressing the endogenous EB1 level decreased the velocity of YFP-Kv1.2 puncta (Fig. 2), and EB1 co-moved with the Kv1.2/Kvβ2 complex at the velocity that is consistent with KIF3-mediated axonal transport (Fig. 3; Figure S4), suggesting a direct role of EB1 in transporting Kv1 channels. The fixation method used here for staining endogenous EB1 was 4% formaldehyde instead of cold methanol, so that the total EB1 level was revealed. EB1 is best known for its plus-end tracking. In this study, we observed a new movement for EB1 when it co-moved with Kv1.2/Kvβ2, which was approximately four times faster than its plus-end tracking (Fig. 3B,C; Figure S4). Although the nature of the movement remains to be determined, our results clearly indicate that unlike many other EB1-binding proteins, neither Kv1.2 nor Kvβ2 undergoes the plus-end tracking movement. Besides binding to microtubule plus ends and seams [31], there is a large pool of EB1 molecules in the cytoplasm. How different pools of EB1 may be involved in regulating kinesin motor functions is an interesting question for future investigation.

Suppressing endogenous KIF3A levels by siRNA markedly decreased anterograde movement of YFP-Kv1.2 puncta (Fig. 2), providing compelling evidence for a critical role of KIF3 in transporting Kv1 channels. Moreover, CFP-Kv1.2 co-moved with KIF3A-YFP along axons (Figure S4E). These results are consistent with our previous study, in which we hypothesized that multimeric Kv1 interaction with dimeric EB1 and KIF3 with two microtubule binding subunits may increase the fidelity of microtubule association and hence transport, analogous to the finding using purified microtubules and yeast proteins [19], [21], [22]. How exactly Kvβ2 stimulated the activities of KIF3 motors in transporting Kv1 channels remains to be determined in future studies. Furthermore, our data do not exclude the possibility that other kinesin motors may be also involved in Kv1 axonal transport. KIF5B was implicated in transporting Kv1.2 channels into axons [32]. To assess the potential role of KIF5B, we constructed KIF5B siRNA plasmids and identified one that significantly suppressed endogenous KIF5B level (approximately 25% reduction). However, different from EB1 and KIF3A siRNAs, the KIF5B siRNA did not significantly alter YFP-Kv1.2 axonal transport (data not shown). Nonetheless, it is possible that KIF5 or other kinesin motors are involved in transport Kv1 channels in certain type of cells or at certain developmental stage.

Surprisingly, Kvβ2 significantly enhanced the retrograde transport of YFP-Kv1.2 puncta (Fig. 1E,G,I). This result has raised an interesting question. How does Kvβ2 increase Kv1.2 axonal targeting by enhancing Kv1.2 axonal transport in both directions? The Kvβ2-mediated enhancement of Kv1.2 axonal transport is biased towards the anterograde direction. For instance, in the presence of expressed Kvβ2, YFP-Kv1.2 puncta moved faster and over longer distance in the anterograde direction compared to the retrograde direction (Fig. 1D–G). Moreover, the moving frequency of YFP-Kv1.2 puncta increased in the anterograde but not retrograde direction (Fig. 1H,I). The increase of retrograde velocity suggests that Kvβ2 may stimulate dynein activity as well. In addition to the possibility that Kvβ2 directly regulates dynein activity, Kvβ2 may regulate the activities of both KIF3 and dynein motors via dynactin, resulting in similar effects on both motors. It has been hypothesized that dynactin can coordinate the activities of kinesin and dynein motors [17], [18]. p150^Glued^, a central component of dynactin, binds to both EB1 and kinesin II [17], [33]. Therefore, understanding how Kvβ2 is linked to dynactin may contribute to elucidating the roles of Kvβ2 in the bi-directional transport of Kv1 channels.

How many Kv1 channel complexes are transported in each vesicle? After calibrating our fluorescence microscope with fission yeast *S. pombe* expressing different YFP-fusion proteins [29], [30], we measured the fluorescence intensity of each YFP-Kv1.2 punctum and estimated that there were approximately 23 YFP-Kv1.2 channel tetramers in an anterograde punctum on average, 27 in a retrograde punctum, and 70 in a stationary punctum. The caveats for these numbers are that (1) the micro-environment of different proteins and cells may not significantly impact the YFP fluorescence, (2) over-expressed YFP-Kv1.2 may be enriched into transport vesicles similar to the native Kv1 channels. Nonetheless, this study represents the first attempt to estimate the potassium channel number in transporting vesicles along axons. Taken together, this study shows that proper type and amount of Kv1 associated proteins are critical for the dynamics of Kv1 transporting puncta. Owing to potential different associated proteins [8], [9], [19], [28], [34], Kv channel axonal transport may differ for different types of channels and in different types of neurons. Moreover, it can be regulated during development and by neuronal activity. There are interesting topics for future studies. Therefore, this study lays a solid foundation for quantitatively understanding developmental and activity-dependent regulation of Kv channel trafficking in axons.

## Supporting Information

Movie S1 YFP-Kv1.2 puncta moving anterogradely in the presence of co-expressed Kv β 2. YFP-Kv1.2 puncta moved anterogradely along the proximal region of an axon shown in Fig. 1B. The neuron was transfected with YFP-Kv1.2 and Kv β 2 at 5 DIV, and imaged 2 days later with the FRAP strategy. The left side is towards soma and the right side towards axonal growth cone. Signals are inverted. The total time is 198 seconds (s) and the total length is 78 µm.
(5.97 MB AVI)

Movie S2 YFP-Kv1.2 puncta moving retrogradely in the presence of co-expressed Kv β 2. YFP-Kv1.2 puncta moved retrogradely along the proximal region of an axon as shown in Fig. 1C. The left side is towards soma and the right side towards axonal growth cone. The total time is 198 s and the total length is 46 µm.
(2.00 MB AVI)

Figure S1 Visualization and quantification of mobile puncta containing YFP-Kv1.2 in axons (A) The procedure of FRAP imaging. A neuron expressing YFP-Kv1.2 was chosen for the FRAP imaging shown by camera lucida drawing (left) with dendrites in red and the axon in blue. The YFP fluorescence in a segment of the proximal axon was first bleached with maximal excitation lights, indicated by a dashed circle (middle). Next, time-lapse imaging was performed with weak excitation lights after bleaching (right). The black cornered area is shown in Fig. 1B and the red cornered area is shown in Fig. 1C. (B) Kymographs of anterograde (left) and retrograde (right) puncta of YFP-Kv1.2 in the absence of co-expressed Kv β 2. (C) Kymographs of anterograde (left) and retrograde (right) puncta of YFP-Kv1.2 in the presence of co-expressed Kv β 2. These kymographs were generated from images captured from the proximal (within 150 µm from the soma), middle, and distal (within 150 µm from the axonal growth cone) regions of axons. (D) Measurement and calculation of the travel distance (d), the moving time (t_m_), the stationary time (t_s_), the velocity (v), the percentage of moving time (P_moving_), and the mobile punctum frequency (F).
(6.89 MB TIF)

Figure S2 Suppressing endogenous EB1 and KIF3A levels by vector-based siRNA (A) EB1 siR2 with mCherry (red) as the indicator knocked down endogenous EB1 (green). (B) KIF3A siR2 (red) knocked down endogenous KIF3A (green). Neurons were transfected with siRNA plasmids at 4 DIV, and fixed and stained at 8 DIV. The statistics are shown in Fig. 2A.
(9.73 MB TIF)

Figure S3 Neurons co-transfected with YFP-Kv1.2, Kv β 2 and siRNA vectors Hippocampal neurons were transfected at 4 DIV, fixed and stained at 8-9 DIV. Signals are inverted. Neurons co-transfected with YFP-Kv1.2 (green in the merged), Kv β 2, and either control siRNA (A) or EB1 siR2 (B) plasmids (red in the merged), were stained for endogenous EB1 (blue in the merged). Neurons co-transfected with YFP-Kv1.2, Kv β 2, and either control siRNA (C) or KIF3A siR2 (D) plasmids, were stained for endogenous KIF3A (blue in the merged). Arrows, axons; Arrowheads, dendrites. The boxed areas are enlarged for four times and shown in Fig. 2B. Scale bars, 100 µm.
(9.82 MB TIF)

Figure S4 Co-movement of fluorescently-tagged Kv β 2/EB1 and Kv1.2/KIF3A (A) A kymograph for EB1-YFP plus-end tracking. (B) A kymograph for YFP-Kv β 2. (C) Anterograde co-movement of EB1-YFP and CFP-Kv β 2. (D) Retrograde co-movement of EB1-YFP and CFP-Kv β 2. Blue arrowheads, EB1 plus-end tracking. Red arrow, anterograde co-movement. Red arrowheads, retrograde co-movement. (E) Anterograde and retrograde co-movement of CFP-Kv1.2 and KIF3A-YFP.
(10.27 MB TIF)
