# Supplementary figures and images for: Dynamics of Kv1 Channel Transport in Axons
Source: PLoS One. 2010 Aug 4;5(8):e11931. doi: 10.1371/journal.pone.0011931 (PMC2915926; doi:10.1371/journal.pone.0011931)

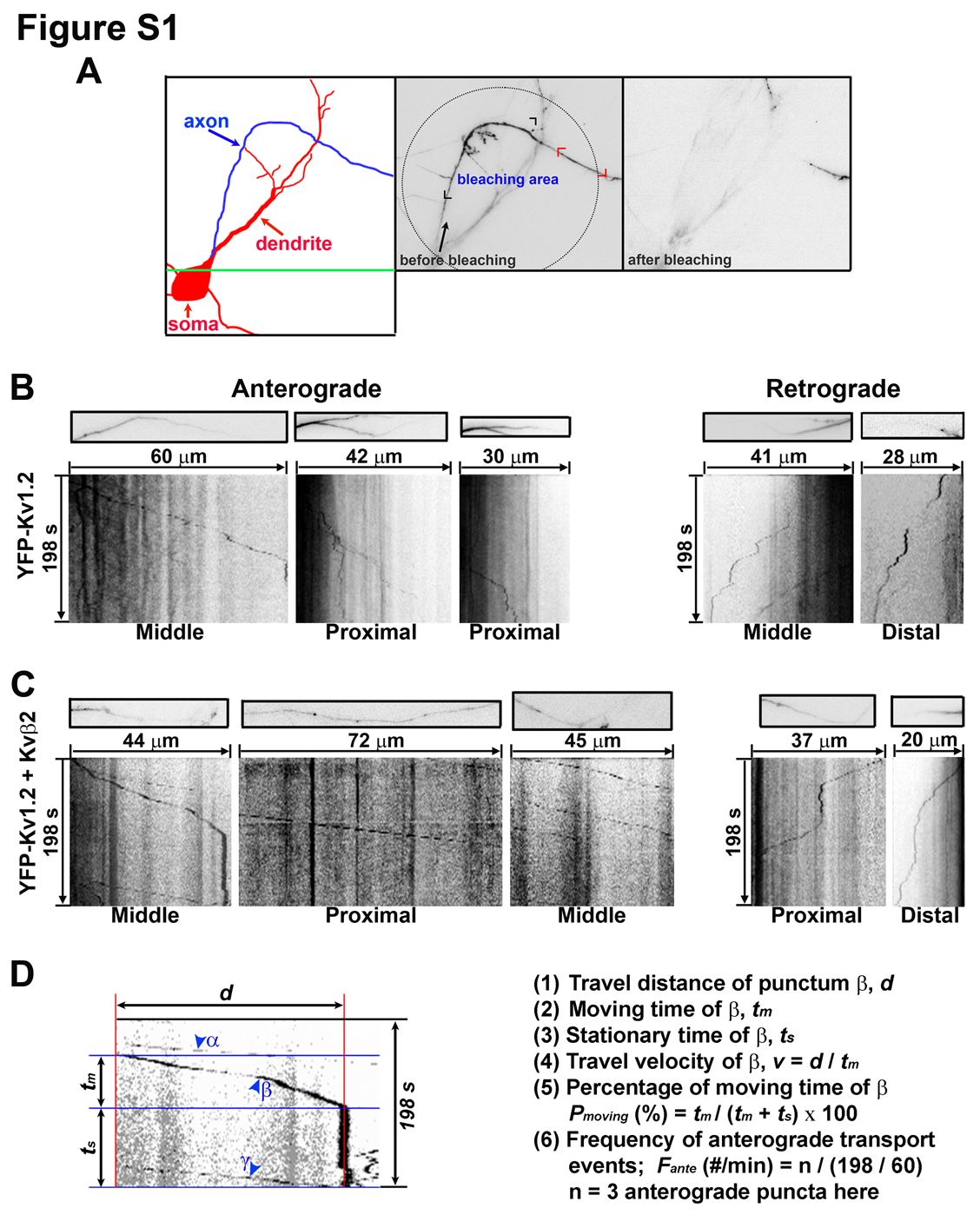

Supplement: Figure S1 — Visualization and quantification of mobile puncta containing YFP-Kv1.2 in axons (A) The procedure of FRAP imaging. A neuron expressing YFP-Kv1.2 was chosen for the FRAP imaging shown by camera lucida drawing (left) with dendrites in red and the axon in blue. The YFP fluorescence in a segment of the proximal axon was first bleached with maximal excitation lights, indicated by a dashed circle (middle). Next, time-lapse imaging was performed with weak excitation lights after bleaching (right). The black cornered area is shown in Fig. 1B and the red cornered area is shown in Fig. 1C. (B) Kymographs of anterograde (left) and retrograde (right) puncta of YFP-Kv1.2 in the absence of co-expressed Kv β 2. (C) Kymographs of anterograde (left) and retrograde (right) puncta of YFP-Kv1.2 in the presence of co-expressed Kv β 2. These kymographs were generated from images captured from the proximal (within 150 µm from the soma), middle, and distal (within 150 µm from the axonal growth cone) regions of axons. (D) Measurement and calculation of the travel distance (d), the moving time (tm), the stationary time (ts), the velocity (v), the percentage of moving time (Pmoving), and the mobile punctum frequency (F). (6.89 MB TIF) [file pone.0011931.s003.tif]

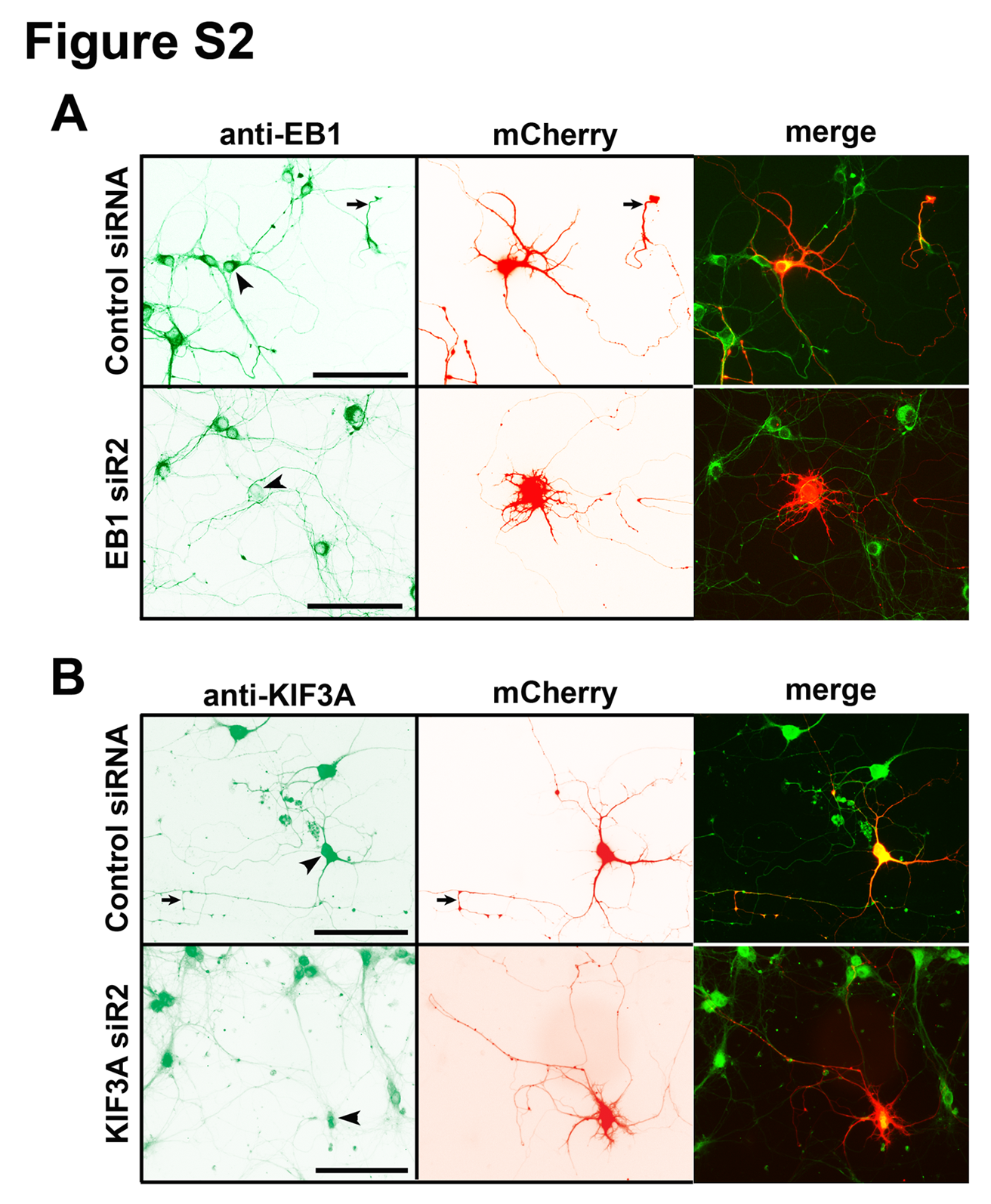

Supplement: Figure S2 — Suppressing endogenous EB1 and KIF3A levels by vector-based siRNA (A) EB1 siR2 with mCherry (red) as the indicator knocked down endogenous EB1 (green). (B) KIF3A siR2 (red) knocked down endogenous KIF3A (green). Neurons were transfected with siRNA plasmids at 4 DIV, and fixed and stained at 8 DIV. The statistics are shown in Fig. 2A. (9.73 MB TIF) [file pone.0011931.s004.tif]

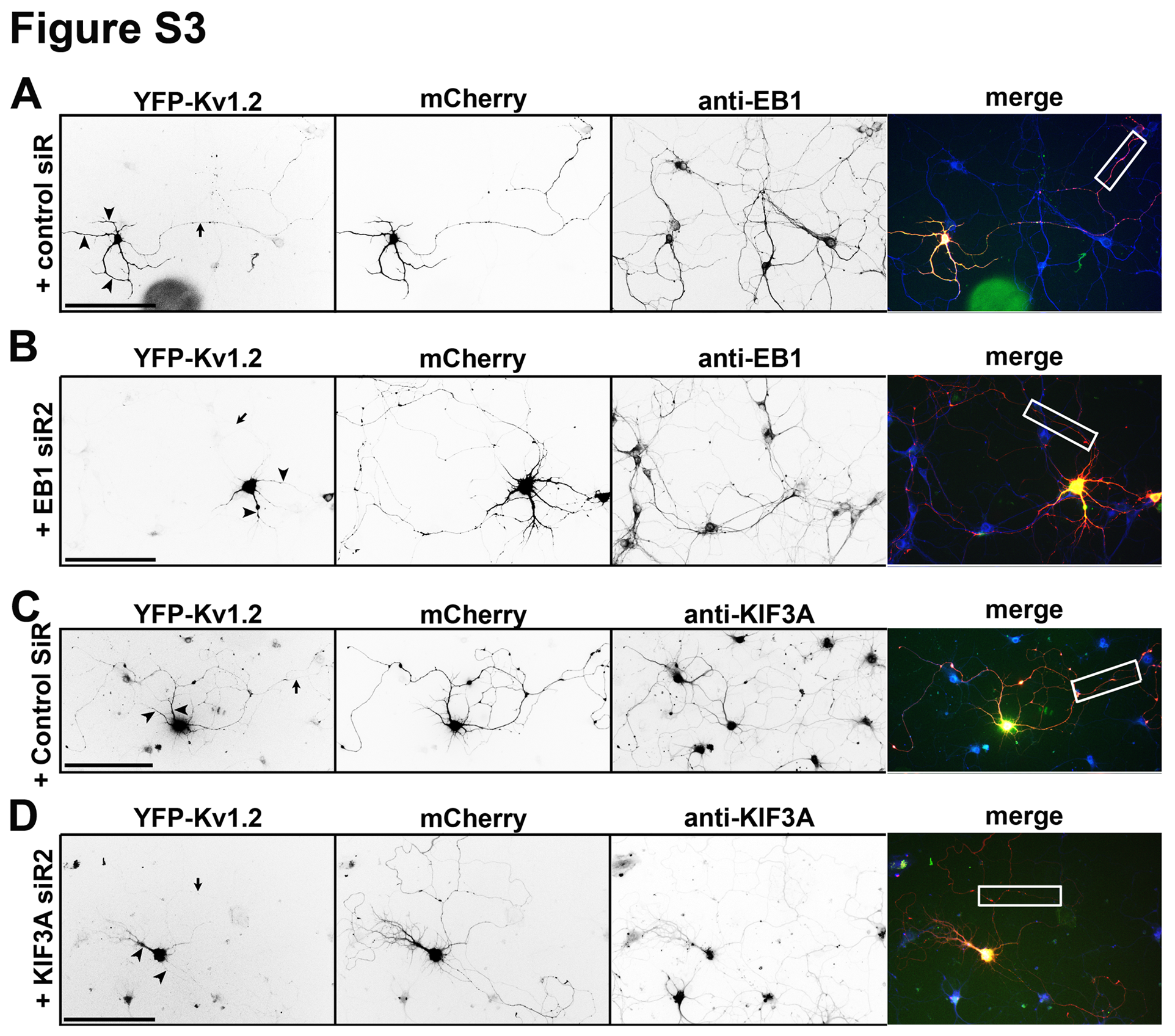

Supplement: Figure S3 — Neurons co-transfected with YFP-Kv1.2, Kv β 2 and siRNA vectors Hippocampal neurons were transfected at 4 DIV, fixed and stained at 8-9 DIV. Signals are inverted. Neurons co-transfected with YFP-Kv1.2 (green in the merged), Kv β 2, and either control siRNA (A) or EB1 siR2 (B) plasmids (red in the merged), were stained for endogenous EB1 (blue in the merged). Neurons co-transfected with YFP-Kv1.2, Kv β 2, and either control siRNA (C) or KIF3A siR2 (D) plasmids, were stained for endogenous KIF3A (blue in the merged). Arrows, axons; Arrowheads, dendrites. The boxed areas are enlarged for four times and shown in Fig. 2B. Scale bars, 100 µm. (9.82 MB TIF) [file pone.0011931.s005.tif]

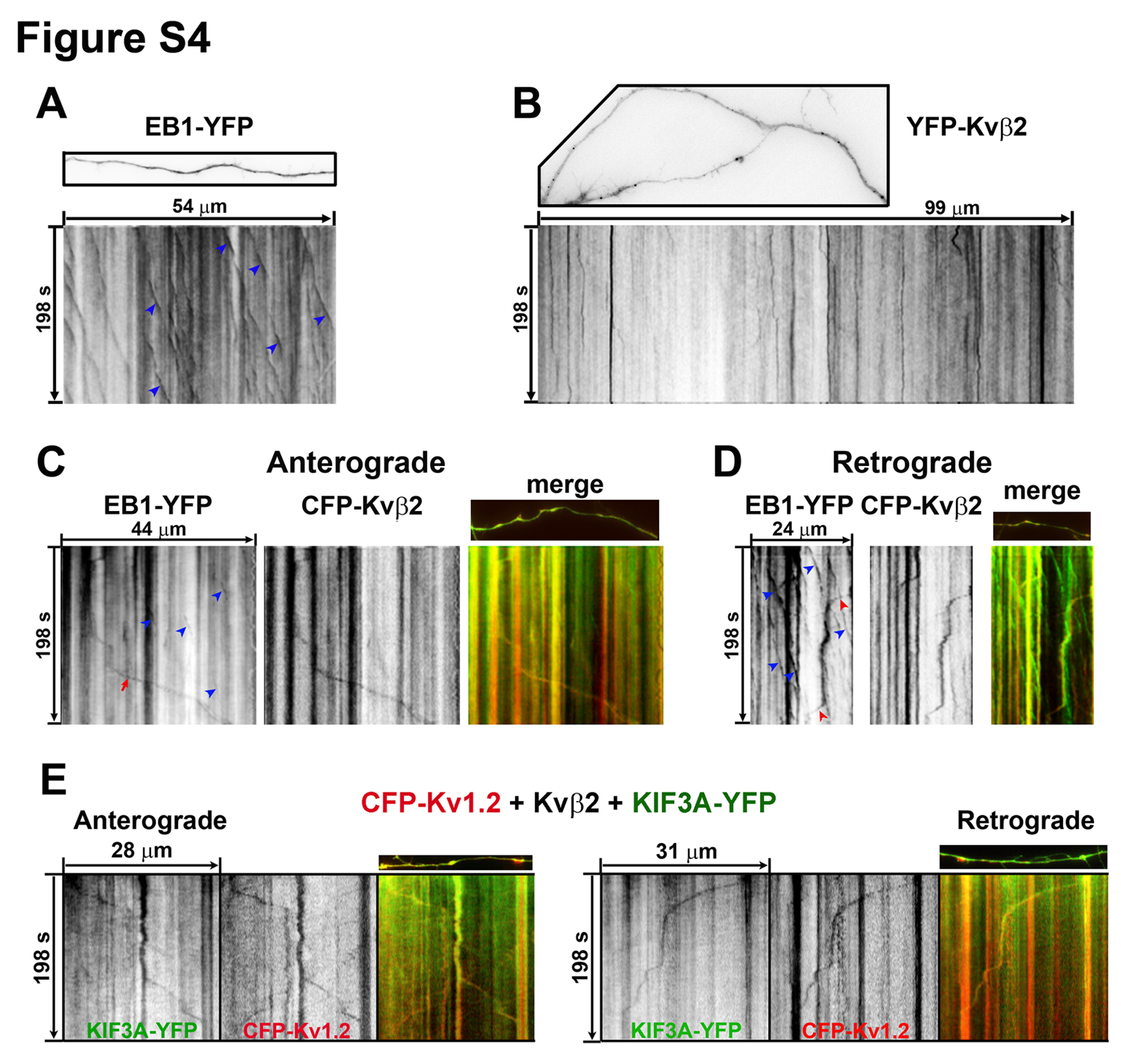

Supplement: Figure S4 — Co-movement of fluorescently-tagged Kv β 2/EB1 and Kv1.2/KIF3A (A) A kymograph for EB1-YFP plus-end tracking. (B) A kymograph for YFP-Kv β 2. (C) Anterograde co-movement of EB1-YFP and CFP-Kv β 2. (D) Retrograde co-movement of EB1-YFP and CFP-Kv β 2. Blue arrowheads, EB1 plus-end tracking. Red arrow, anterograde co-movement. Red arrowheads, retrograde co-movement. (E) Anterograde and retrograde co-movement of CFP-Kv1.2 and KIF3A-YFP. (10.27 MB TIF) [file pone.0011931.s006.tif]
